# Assessing Mathematical Models of Influenza Infections Using Features of the Immune Response

**DOI:** 10.1371/journal.pone.0057088

**Published:** 2013-02-28

**Authors:** Hana M. Dobrovolny, Micaela B. Reddy, Mohamed A. Kamal, Craig R. Rayner, Catherine A. A. Beauchemin

**Affiliations:** 1 Department of Physics and Astronomy, Texas Christian University, Fort Worth, Texas, United States of America; 2 F. Hoffmann-La Roche Inc., Nutley, New Jersey, United States of America; 3 Roche Products Pty Ltd. and Faculty of Pharmacy and Pharmaceutical Sciences, Monash University, Melbourne, Australia; 4 Department of Physics, Ryerson University, Toronto, Ontario, Canada; Albert Einstein College of Medicine, United States of America

## Abstract

The role of the host immune response in determining the severity and duration of an influenza infection is still unclear. In order to identify severity factors and more accurately predict the course of an influenza infection within a human host, an understanding of the impact of host factors on the infection process is required. Despite the lack of sufficiently diverse experimental data describing the time course of the various immune response components, published mathematical models were constructed from limited human or animal data using various strategies and simplifying assumptions. To assess the validity of these models, we assemble previously published experimental data of the dynamics and role of cytotoxic T lymphocytes, antibodies, and interferon and determined qualitative key features of their effect that should be captured by mathematical models. We test these existing models by confronting them with experimental data and find that no single model agrees completely with the variety of influenza viral kinetics responses observed experimentally when various immune response components are suppressed. Our analysis highlights the strong and weak points of each mathematical model and highlights areas where additional experimental data could elucidate specific mechanisms, constrain model design, and complete our understanding of the immune response to influenza.

## Introduction

The Centers for Disease Control and Prevention estimate that in the United States deaths related to influenza ranged from about 3,000 to 49,000 deaths per season from the 1976/77 to the 2006/07 flu seasons [1]. While virologists, microbiologists, and clinicians have studied the influenza virus and the illness it causes for many years, it is only relatively recently that mathematical modelling has been used to provide insight into influenza infections [2], [3].

Application of mathematical modelling holds great promise and the analysis of various experimental data has furthered our understanding of influenza. Models have been used to quantitatively determine key influenza kinetic parameters such as the duration of the eclipse phase and the viral clearance rate [4], [5]. They have also been used to optimize antiviral therapy regimens, better characterize antiviral efficacy, and predict the emergence of drug resistance [5]–[8]. Mathematical models of within-host influenza infections can provide unique and valuable insights, but they must correctly capture the dynamics of the disease for full utility.

One major obstacle to creating a biologically accurate model of influenza infections has been the incorporation of a biologically realistic immune response. An accurate model of the key players of the immune response is essential to capture the range of dynamics of influenza infections particularly since the immune response is thought to play an important role in eliminating the infection [9]–[11]. Immune memory or strength of the immune response is also believed to play an important role in shaping the severity of an influenza infection [12]–[16]. Unfortunately, study of the host immune response to influenza suffers from a paucity of data describing the dynamics of both the adaptive and innate immune responses during infection. Data from human patients are typically for few time points [17]–[20]. Animal experiments are sometimes more comprehensive [11], [21]–[25], capturing levels of various cytokines/chemokines [11], [21], [25] and immune cells [22]–[24] at several time points. However, the immune response in animals is known to differ from that in humans [26]–[29], particularly in Balb/c mice, a popular experimental model lacking functional expression of Mx, an IFN-induced protein that induces an antiviral state in cells [29], [30]. Deficiencies in data limit the formulation of a comprehensive, quantitative picture of the immune response to influenza.

In this context, mathematical modelling can provide valuable insights and help guide investigation. Already, several mathematical models for the course of an influenza infection within a host have incorporated an immune response [2], [4], [22], [23], [31]–[36]. They range from simple models that primarily aim to resolve the effects of a few specific components of the host immune response using simplifying assumptions [4], [23], [32]–[37] to complicated models with many equations and parameters describing the detailed interactions of immune response components [2], [22], [31]. Unfortunately, since viral titer is often the only experimental quantity measured over time, even adding a simple immune response with limited additional parameters can be problematic as it becomes difficult to ascertain biologically realistic parameters for the models [38].

Here, we amass previously published experimental and clinical data on the time course and impact of various immune components. These data are used to construct a picture of the role of three key immune response components: antibodies (Abs), cytotoxic T lymphocytes (CTLs), and interferon (IFN). We also assemble a set of published mathematical models of influenza infections that contain an explicit immune response. We confront them with the experimental data to assess how well they reproduce the time course of the immune response and the effect of individual immune components on the viral titer. We quantitatively assess the relative contributions of Abs, CTLs, and IFN by measuring their individual effect on various characteristics of the influenza infection and we investigate the effect of antiviral therapy in the presence and absence of an immune response. Our analysis identifies key qualitative features of the immune response to influenza that must be incorporated in mathematical models in order for these models to serve as surrogates to experimental investigation as valuable, credible influenza infection simulators and predictors.

## Results

### Experimental evidence for the role of the immune response

The course of an influenza infection within a host is typically probed using viral titer time courses, sometimes supplemented by symptom scores, cytokine concentration time courses, and rare measurements of Ab levels on one or two days [18], [20], [39]–[42]. While this approach enables the identification of correlations between immune factors and infection severity markers, it rarely establishes causal relationships. Ideally, the characterization of the role and efficacy of a specific immune response component would follow an approach similar to that used for characterizing antiviral efficacy: dose-ranging experiments in which increasing concentrations of the antiviral are applied to isolate and quantify its effect on the infection course. While such a procedure is virtually impossible to perform for immune response components such as Abs or CTLs, knock-out or suppression experiments in animals and the occurrence of natural infections in immunocompromised patients provide similar conditions. These types of studies provide compelling evidence for the relative role and importance of the host immune response in the control of influenza infections.

#### Clinical data

**Figure 1:**
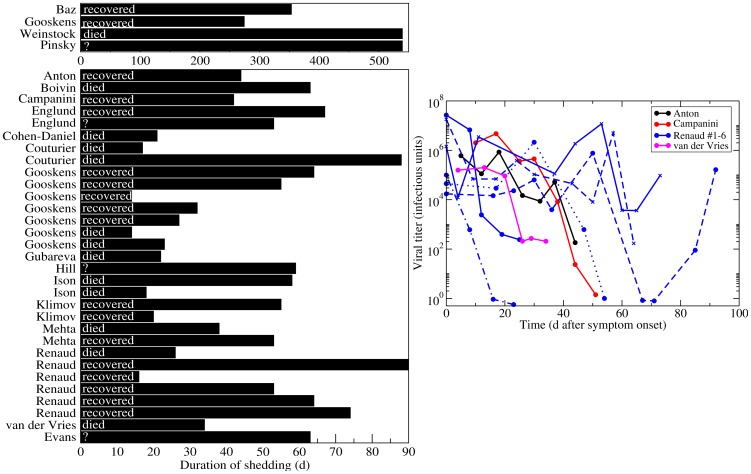
Duration of viral shedding in immunocompromised patients. (Left) The reported duration of viral shedding for individual immunocompromised patients infected with influenza as chronicled in the literature is indicated by bar length. Infection outcome is indicated (died or recovered) when known, or labelled as unreported with ‘?’. (Right) Viral titer time courses of immunocompromised patients infected with influenza. Since all patients were treated with various courses of antivirals and given the prolonged shedding in these patients, recovery is most likely due to the eventual success of antiviral therapy rather than to clearance of the infection by the limited host immune response.

The infection of immunocompromised individuals provides a window into the course of the infection when the immune response is severely attenuated. In many reported cases of immunocompromised patients with influenza, virus was shed for long periods of time, i.e., in some cases more than a year [43]–[60], (Fig. 1). Underlying medical conditions, the influenza viral strain, and antivirals used to treat immunocompromised patients are given in Table 1.

**Table 1:** Details of infections of immunocompromised patients.

Paper	Medical condition	Strain	Antiviral treatment 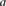
Antón [47]	chronic lymphocytic leukemia	A/pH1N1 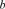	ost, zvr
Baz [43]	SCID 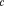 (SCT) 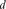	A/H3N2	ost, amt, zvr
Boivin [48]	chronic lymphoid leukemia	A	amt, rbv
Campanini [49]	acute lymphoid leukemia	A/H1N1	ost
Cohen-Daniel [51]	acute monocytic leukemia (SCT)	A/H3N2	ost
Couturier [52] (case 1)	multiple myeloma (SCT)	A/pH1N1	ost, zvr
Couturier [52] (case 2)	multiple myeloma (SCT)	A	ost, zvr
CDC [50] (case 1)	leukemia (SCT)	A/pH1N1	ost
CDC [50] (case 2)	leukemia (SCT)	A/pH1N1	ost, rmt, zvr, rbv
Evans [60]	Human immunodeficiency virus (HIV)	A	rmt
Gooskens [44] (case 1)	SCID	A/H3N2	ost
Gooskens [44] (case 2)	Non-Hodgkin lymphoma (SCT)	A/H3N2	ost
Gooskens [44] (case 3)	Non-Hodgkin lymphoma	A/H3N2	none
Gooskens [44] (case 4)	Anaplastic large cell lymphoma (SCT)	B	none
Gooskens [44] (case 5)	Non-Hodgkin lymphoma	A/H3N2	none
Gooskens [44] (case 6)	Non-Hodgkin lymphoma (SCT)	A/H1N1	none
Gooskens [44] (case 7)	Acute myelogenous leukemia (SCT)	B	none
Gooskens [44] (case 8)	Acute myelogenous leukemia (SCT)	A/H1N1	none
Gubareva [53]	chronic myelocytic leukemia	B	rbv, zvr
Hill-Cawthorne [54]	Hodgkin lymphoma (SCT)	A/pH1N1	ost, zvr
Ison [55] (case 1)	myelomonocytic leukemia (SCT)	B	ost, rbv
Ison [55] (case 2)	chronic lymphocytic leukemia	A/H3N2	ost, rmt, zvr
Klimov [56] (case 1)	SCID (SCT)	A/H3N2	amt
Klimov [56] (case 2)	acute myelogenous leukemia (SCT)	A/H3N2	amt
Mehta [57] (case 1)	myelodysplastic syndrome (SCT)	A/pH1N1	ost, pvr
Mehta [57] (case 2)	acute myelogenous leukemia	A/H1N1	ost, pvr, zvr
Pinsky [46]	Wiskott-Aldrich syndrome (SCT)	A/H1N1	unknown
Renaud [58] (case 1)	acute myeloblastic leukemia (SCT)	A/pH1N1	ost, pvr, rbv, rmt, zvr
Renaud [58] (case 2)	acute myeloblastic leukemia (SCT)	A/pH1N1	ost, rbv, rmt, zvr
Renaud [58] (case 3)	chronic myeloblastic leukemia (SCT)	A/pH1N1	ost
Renaud [58] (case 4)	acute lymphoblastic leukemia	A/pH1N1	ost, zvr
Renaud [58] (case 5)	Hodgkin lymphoma	A/pH1N1	ost, pvr, rbv, rmt
Renaud [58] (case 6)	acute lymphoblastic leukemia (SCT)	A/pH1N1	ost
van der Vries [59]	chronic lymphocytic leukemia	A/H1N1	ost, amt, zvr
Weinstock [45]	acute lymphocytic leukemia	A/H1N1	ost, amt, zvr, rmt

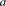
 Antiviral abbreviations are as follows: oseltamivir (ost), amantadine (amt), rimantadine (rmt), ribavarin (rbv), zanamivir (zvr), peramivir (pvr).

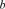
 A/pH1N1: 2009 pandemic H1N1.

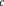
 SCID: Severe combined immunodeficiency disease.

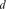
 SCT: Stem cell transplant.

The average duration of viral shedding for uncomplicated seasonal influenza infections in healthy adults is 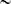
5 d [61]. In contrast, immunocompromised patients shed virus for at least 14 d, with two reports of patients shedding virus for at least 18 months. Viral loads in the immunocompromised typically remain high and fairly constant for the duration of the infection, despite treatment with antivirals, indicating that the immune response is an important component to clearing influenza infection in humans. Unfortunately, the data presented in these reports do not allow us to assess whether the lack of an immune response leads directly to chronic infections or whether it causes a more complex infection, such as an infection that spreads beyond the upper respiratory tract [62], [63] or one that includes a bacterial co-infection [64], [65], that is simply more difficult to clear even with antiviral therapy.

#### Preclinical data

**Figure 2:**
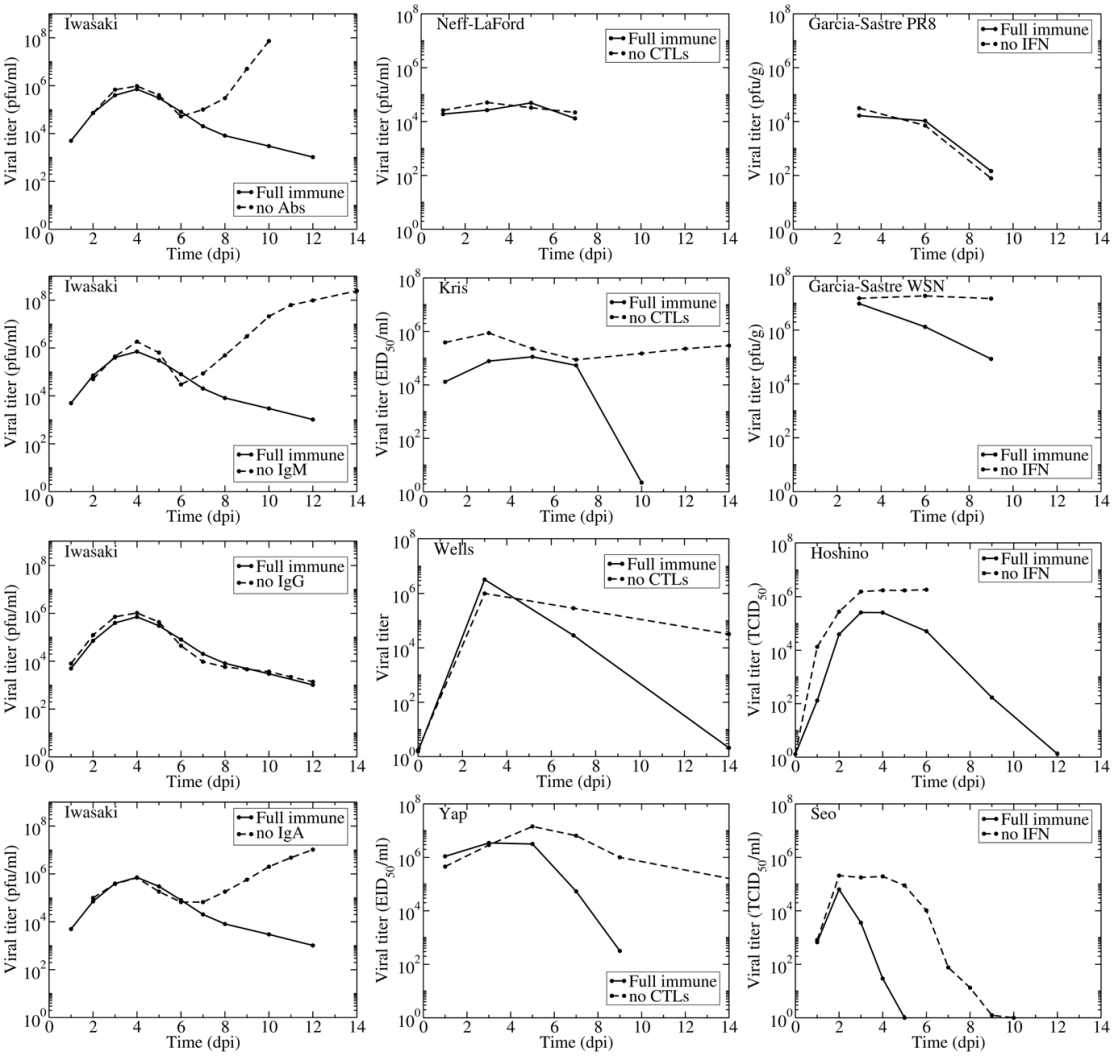
Experimental evidence for the effect of Abs, CTLs, and IFN. Published viral titer time courses for influenza infection in normal (solid) or immunocompromised (dashed) mice (or pigs in Seo) where either the Abs (left column), CTLs (centre column), or IFN (right column) responses were individually compromised by various means (e.g., toxins, antisera, knock-out). Data extracted from Iwasaki [11], Neff-LaFord [70] Kris [66], Wells [67], Yap [118], Garcia-Sastre [68], Hoshino [25], and Seo [13].

In animal models, the effect of individual components of the immune response on the course and outcome of the infection can be evaluated by down-regulating or neutralizing a specific component of the immune response or its activity. These studies use component-specific antisera [11], [25], genetic modification (knock-out) [13], [66]–[69], or treatment with toxins [70]. Unfortunately, these manipulations sometimes do not fully remove the immune component [25], [67], [71] or removal of the component also affects other components of the immune response [11], [72]. Additionally, the immune response in these animal models might not be representative of that of a human host [28], [29]. Despite these shortcomings, experiments in animals can help shape our understanding of the relative role of different immune components. Published results of these experiments conducted in mice (with the exception of Seo [13], which was conducted in pigs) are assembled in Fig. 2 where graphs show the viral titer time course for an influenza infection with a full immune response or with one immune component disabled or destroyed.

We focused on published experimental data in which the authors had disabled one or more of the following immune responses: cytotoxic T lymphocytes (CTLs) which kill infected cells [73], [74]; antibodies (Abs) which bind and inactivate virus [75]–[77]; and interferon (IFN) which has many modes of action including reducing the production of virus by infectious cells and establishing an antiviral state in susceptible cells rendering them resistant to infection [78]–[80].

For Abs, we found only one experiment with complete viral time courses with and without various Ab isotypes. Iwasaki et al. used either antisera to disable individual Ab isotypes (IgM, IgG, and IgA) or B-cell depleted mice to disable all Abs [11]. In addition to the viral titers shown in Fig. 2, Iwasaki et al. measured time courses of each Ab isotype and IFN levels, but not CTL levels. The thorough measurements made in this remarkable experiment clearly establish that the antisera depleted Abs below detection level while leaving the IFN response unaffected (not shown here but appearing in the original publication). The viral titer time courses show that Abs, specifically IgA and IgM Abs, begin affecting viral titer levels around 6 days post-infection (dpi). IgG Abs appear to have no effect on the course of the infection. In the presence and absence of an Ab response, the viral titer first peaks around 4 dpi after which it decreases slightly. At 6 dpi the effect of Abs becomes apparent — without Abs viral titer increases rapidly from this point, but with Abs there is a continued decline in viral titer. These experiments indicate Abs play a critical role in terminating the infection although they do not significantly affect the initial viral titer peak.

Several experiments have studied the role of CTLs in influenza infections. Three of the experiments presented here (Kris, Wells, and Yap) use nude mice, which have a deteriorated or absent thymus [66], [67], [69]. The remaining experiment by Neff-LaFord et al. used TCDD, a toxin that depletes CD8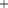 T cells [70]. Neither of these methods completely eliminates the CTL response; nude mice have at least 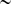
1,000-fold lower CTL levels in their lungs compared to normal mice [67] while the TCDD-treated mice have CTL levels 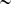
15% that of untreated mice [71]. An additional concern with these experiments is that the depletion of CTLs can also affect the amount of IFN [11], [72] since CTLs are in part responsible for producing the IFN-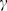 response [81]. It is also known that nude mice have functionally impaired B cells [82] which could lead to an inadequate Ab response in these mice. These shortcomings could explain the wider variation in the predicted role of CTLs. Neff-LaFord et al. found no significant difference in viral titers between normal and CTL-depleted mice [70]. This might be because TCDD treatment is not entirely effective at disabling CTLs, or because data were only collected until 7 which might be too early for CTLs to have significantly affected the viral titer time course. The remaining three experiments suggest CTLs begin to affect the viral titer time course anywhere between 5 to 8 dpi. In the absence of CTLs, all three experiments showed the viral titer remaining elevated (but not growing as seen when disabling Abs) for a long period of time. This suggests CTLs play a role in the resolution of the infection but not in controlling viral titer levels. These observations correspond with the immunocompromised patients discussed above who develop chronic influenza infections with relatively constant viral titer levels.

Three experiments studied the effect of disabling IFN on the course of an influenza infection. There are several types of interferon: Type I interferons include IFN-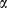, IFN-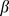, and IFN-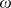 and type II interferons include IFN-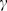. The two types of IFN arise from different genes [83], have different signalling pathways [84], bind to different cell receptors [85] and lead to different antiviral effects [86]. Mathematical models are not yet detailed enough to differentiate between the different types of IFN, and the experiments presented here appear to target both types of IFN, and so we will not differentiate between the different types of IFN for the remainder of the paper.

The three studies on the effect of IFN used different methods to reduce or remove its effect. García-Sastre et al. used mice that had a targeted disruption to the STAT1 gene and as such could not respond to IFN, and performed the experiments with two different influenza A (H1N1) viruses: A/Puerto Rico/8/34 (PR8) and A/Wisconsin/33 (WSN) [68]. Hoshino et al. used an anti-IFN (type not specified) serum to neutralize the IFN response in mice infected with PR8 [25]. Rather than modifying the host response, Seo et al. compared infection with a wild-type PR8 virus to that with a recombinant PR8 virus made resistant to the antiviral effects of IFN via substitution of aspartic (D) with glutamic (E) acid at position 92 of NS1 (D98E) [13]. While Hoshino et al. showed that IFN largely remained below detection level during their experiment, it is not possible to confirm the degree of IFN inhibition in the Garcia-Sastre et al. and Seo et al. experiments as their approach aimed to suppress IFN activity rather than IFN itself [87]–[89]. In the experiments by Hoshino et al. and Seo et al., IFN begins acting early in the infection (1–2 dpi), generally reducing viral load and viral titer peak. This was not the case in the two experiments by Garcia-Sastre et al. which measured no effect on the control of a PR8 infection when the IFN response was disabled, while more sustained titers were observed for WSN infections in the absence of an IFN response. According to Garcia-Sastre et al. the difference in the tissue tropism between the PR8 and WSN strains is responsible for this difference, with PR8 spread being limited by both tissue tropism and the IFN response and WSN spread being limited only by the IFN response. Interestingly, both Garcia-Sastre et al. and Hoshino et al. considered PR8 infections in mice and reported lung homogenate titers, so differences in their results are likely attributable to the different methods used to hinder the IFN response. Overall, the data are not entirely consistent but suggest that the presence of IFN decreases the viral load and that its absence can lead to more sustained viral titer. However, the data were too limited to show whether the absence of an IFN response alone can lead to chronic infection.

### Mathematical models to probe the role of the immune response

Mathematical models are valuable tools in identifying the key players of the immune response against an infection and in resolving their mode of action and efficacy. In constructing a mathematical model for the course of an influenza infection within a host, one carefully considers which host factors to include in the model, which to reduce to a simpler reaction, and which to omit. Each mechanism is implemented in the model based on current knowledge and intuition regarding host-virus interactions. The act of engineering the host-virus interactions into a mathematical model enables us to identify which postulated mechanisms behave inappropriately when implemented and to distill the model down to only those immune response components essential to reproducing general infection kinetics.

**Figure 3:**
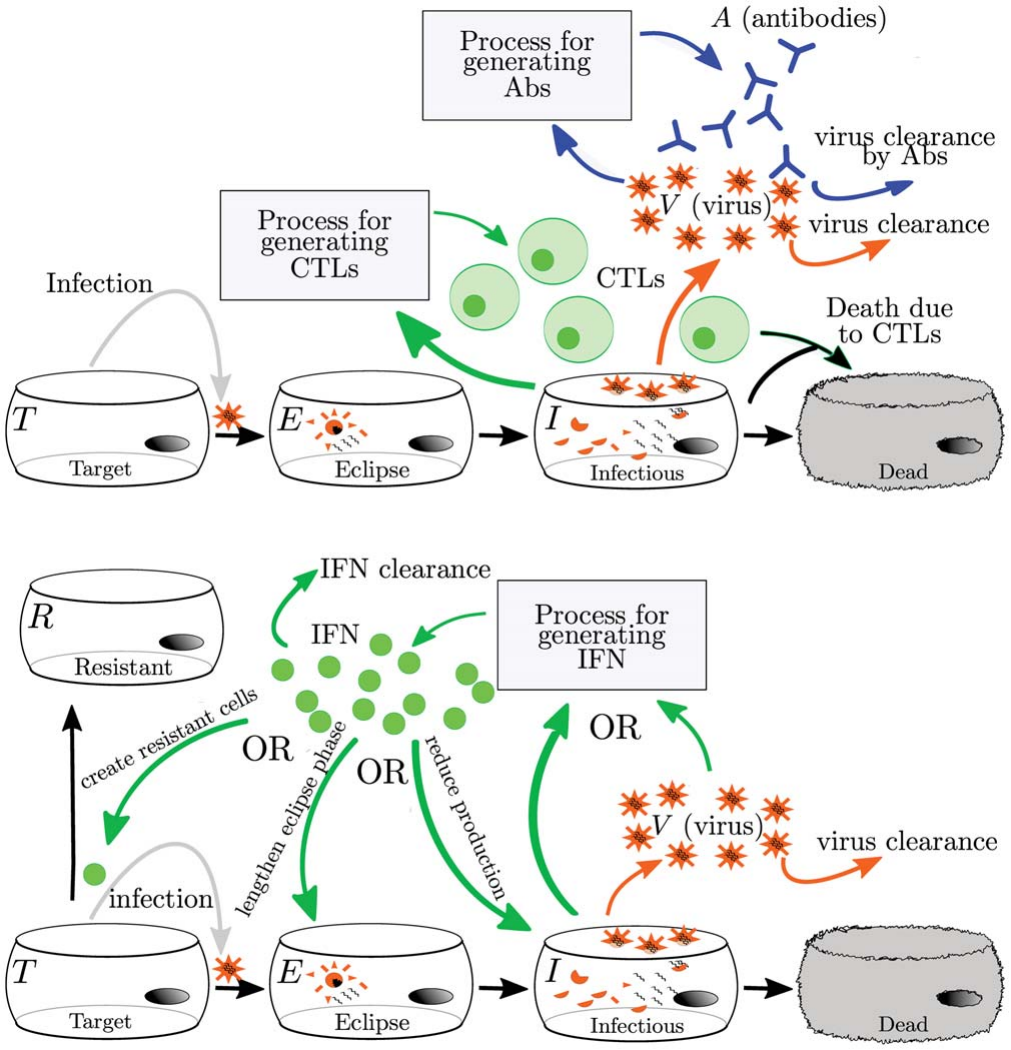
Mathematical models of the adaptive (top) and innate (bottom) immune responses to influenza. The adaptive immune response typically includes Abs and CTLs. The generation of Abs and CTLs are represented with different simplifying assumptions, so the actual processes are not depicted here. All models reviewed here assume that Abs bind to and remove virus while CTLs cause the death of infectious cells. The innate immune response is represented by IFN. Since IFN has many antiviral effects in vivo, models have different implementations of IFN's effect. Details of individual models are discussed in the text and in Supplement S2.

Here, we review eight previously published models of within host influenza infections that explicitly incorporate at least one of the following immune responses: Abs, CTLs, and IFN [2], [4], [6], [22], [23], [31], [32]. We limited our investigation to models with parameters determined from experimental data. A summary of the number of variables, parameters, and immune components incorporated in each model is presented in Table 2, with details provided in supplemental material S2. General schematics of the models' implementation of the adaptive immune response (Abs and CTLs) and the innate immune response (IFN) are shown in Fig. 3. In the case of the Miao et al. model [23], we re-fit their model to their data using an alternative approach and distinguish these two fits of the same model as Miao split (our fit) and Miao full (published). See the Methods section for details.

**Table 2:** Summary of the models presented in this analysis.
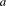

Model	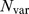	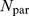	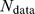	Host	Regen.	Abs/CTL/IFN	Other cells
Bocharov [2]	13	49	19	Human	yes	Abs/CTL/IFN	Antigen-presenting macrophages, T and B helper cells, B cells, plasma cells, IFN-producing macrophages.
Baccam [4]	6	9	36	Human	no	IFN	—
Hancioglu [31]	10	28	0 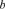	Human	yes	Abs/CTL/IFN	Antigen-presenting macrophages, plasma cells.
Lee [22]	15	48	42	Mouse	yes	Abs/CTL	Dendritic cells, naive and effector CD4 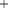 T cells, naive CD8 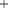 T cells, naive and activated B cells, Long- and short-lived plasma cells.
Handel [6]	6	8	50	Mouse	yes	Abs/IFN	—
Miao [23] 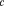	6	8	64	Mouse	yes	Abs/CTL	—
Saenz [32] 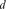	8	12	96	Horse	no	IFN	—
Pawelek [37] 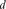	5	11	90	Horse	no	IFN	—

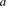
 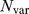 (number of variables), 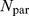 (number of parameters), 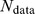 (number of data points), Regen. (whether the model includes cell regeneration).

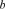
 Model was not mathematically fit to data, but did have to conform to some general criteria.

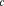
 We consider two different parameter sets for this model (Miao split and Miao full). The differences between these two models are described in Methods.

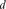
 The Saenz and Pawelek models were fit to the same data.

**Figure 4:**
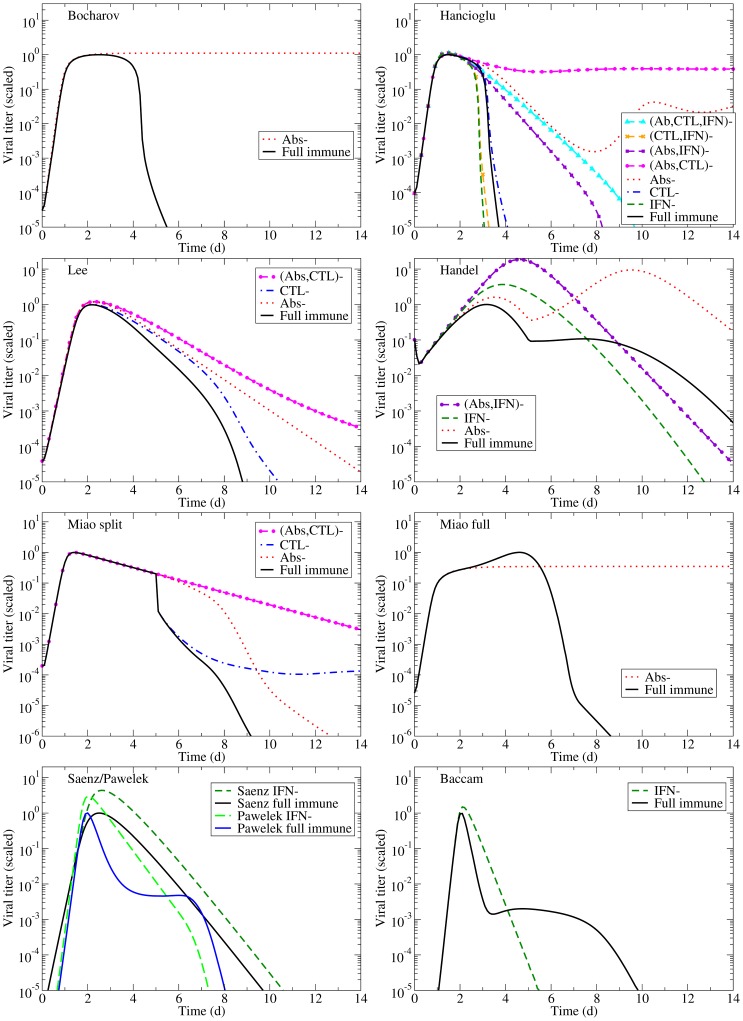
Effects of the immune response on an influenza infection as predicted by mathematical models. The models summarized in Table 2 are used to predict the viral titer time course for influenza infections under various conditions of immune suppression (the ‘–’ sign indicates the component is suppressed). Viral titers for all models have been scaled to peak at 1.0 in the presence of the full immune response to facilitate comparison.

**Figure 5:**
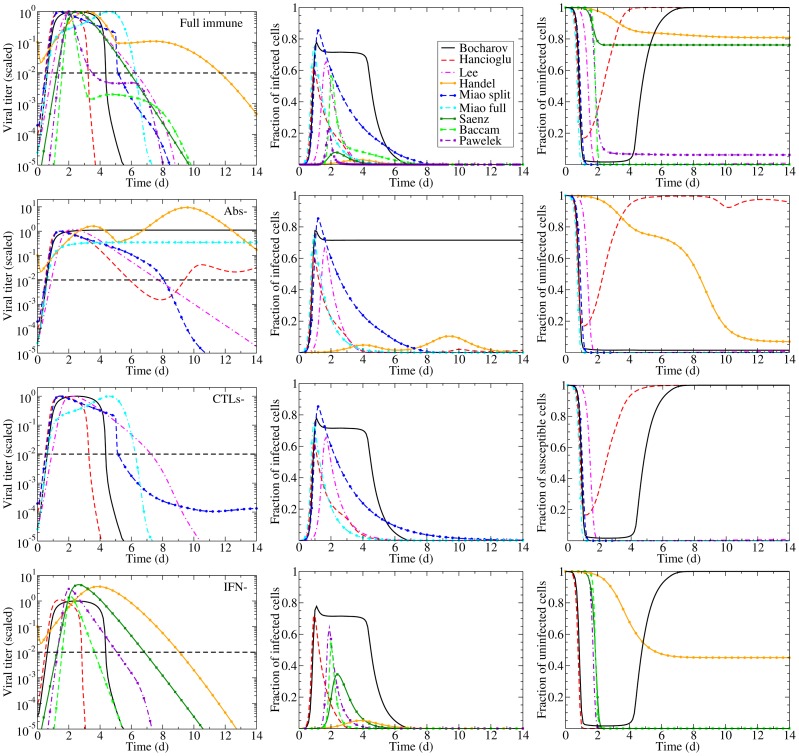
Effect of suppressing Abs, CTLs, or IFN on an influenza infection as predicted by mathematical models. The effect of suppressing each immune component on the time course of the viral titer (left column), and on the fraction of infected (centre column) and uninfected (right column) cells during an influenza infection. The top row illustrates infection kinetics in the presence of a full immune response with subsequent rows showing infection kinetics in the absence of Abs only (second row), CTLs only (third row), or IFN only (bottom row). The dashed lines indicate an approximate symptomatic threshold as defined in [8], [90].

Each of these models can reproduce the experimental infection kinetics against which they were originally validated. By perturbing these models from their basic behaviour, we can test their biological fidelity by determining whether in the presence of immune suppression they reproduce the experimental kinetics presented above. Fig. 4 presents the viral titer time course predicted by each model under various conditions of immune suppression while Fig. 5 explores the predicted effect of suppressing Abs (second row), CTLs (third row), or IFN (fourth row), individually, on the time course of viral titer and fraction of infected and uninfected cells. To facilitate comparison of the behaviour of the various models, all model parameters were scaled so that in the presence of their full immune response, they produce a viral titer time course that peaks at an arbitrary value of 1 (see Methods for details). We assume the infection is symptomatic during any period where the viral titer is above a value of 0.01 (1% of the peak viral titer) as used previously by Dobrovolny et al. [8], [90], and this threshold is indicated by a dashed line in Fig. 5.

The models predict very different viral titer time courses when immune responses are all disabled, ranging from chronic (Fig. 4, Bocharov) or long-lasting (Fig. 4, Miao full) infections, to slowly decaying (Fig. 4, Miao split) or very short-lived (Fig. 4, Baccam) infections. In almost all cases, the infection is target-cell limited, which means that the infection resolves because all cells have been infected and have died (Fig. 5, left column). The exceptions are the Bocharov model, which predicts a chronic infection, and the Handel model, in which a small fraction of cells (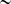
3%) are preserved. The finite number of target cells in these models means that viral growth will always be bounded with the upper limit on the number of viruses given by 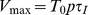, where 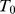 is the initial number (or maximum number) of cells available for infection, 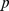 is the production rate and 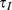 is the average lifespan of an infectious cell. Each target cell, once infected can only produce a finite number of viral particles during its infectious lifespan. As long as the number of target cells remains bounded, which is the case in these models, virus will also remain bounded.

In the absence of an immune response, the viral titer time course should resemble that seen in immunocompromised patients, namely, sustained high viral titers that last for weeks or even months, as discussed above. Instead, most of the mathematical models predict the infection will resolve without the aid of an immune response within a week (Fig. 4). The exceptions are the two Miao models, which produce long-lasting high viral titers, and the Bocharov model, which predicts a true chronic infection. In Miao full, the long-lasting infection is due to poor viral clearance such that while the infection has resolved, the ineffective viral clearance leads to high levels of lingering free virus. In Miao split, it is due to the somewhat long average lifespan of infected cells (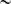
2 d). Unfortunately, neither of these mechanisms can cause the weeks-long, sustained, high viral titers observed in immunocompromised patients. The Bocharov model seems to provide the best representation of an infection in an immunocompromised patient — the chronic infection persists because cell regeneration provides an endless supply of susceptible cells that allow the infection to persist. It is also important to note that completely disabling the immune response in these models does not correspond to the infection progression in the absence of an immune response. Instead, it corresponds to an infection in which the immune response components explicitly included in the model (e.g., IFN, CTLs, Abs) have been disabled. For example, disabling Abs and IFN in the Handel model, which does not explicitly include CTLs, leaves the CTL response intact because the model implicitly takes it into account via the remaining infection parameters of the model (such as the lifespan of infected cells).

**Figure 6:**
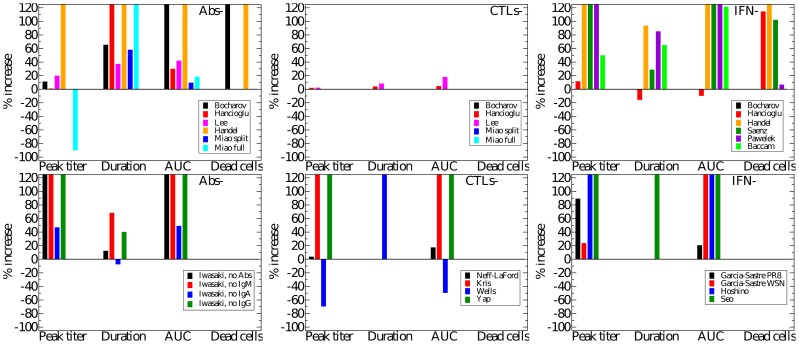
Immune kinetics and its effect on disease severity. The percent increase in peak viral titer, symptom duration, AUC of viral titer, and proportion of dead cells when the effect of Abs (left), CTLs (centre), or IFN (right) is removed in various mathematical models (colour-coded) of influenza infections (top row) or from experimental data (bottom row). Note that a negative percent increase for a given measure indicates a *decrease* of that measure in the absence of that immune response.

Going beyond the qualitative changes to the viral titer time course brought about by Abs, CTLs, and IFN, Fig. 6 offers a quantitative analysis of their relative contribution to decreasing various measures related to the severity of the infection in a patient. For example, we take the peak viral titer to be an approximate measure of the degree of dissemination of the virus within the patient with higher viral loads representing a more disseminated infection. The duration of the symptomatic infection, measured here as the time spent by the viral titer curve over a titer of 0.01 (i.e., above 1% of its peak value, as used in [8], [90]), gives a measure of infection duration and helps distinguish short-lived seasonal infections from more severe or chronic infections. The area under the viral titer curve (AUC) is related to the total amount of virus shedding, and so can be linked to the person-to-person transmission rate of the infection [7], [61], [91]. Finally, the fraction of dead cells at the end of the infection measures the amount of epithelium destruction caused by the infection and can be used to assess the severity of the infection. Together, these measures provide an overview of the infection course which we use to assess how effective various immune responses are in modulating infection severity and patient outcomes. Fig. 6 presents the percent increase in each severity measure that results from the suppression of either Abs, CTLs, or IFN as determined from the experimental data (bottom row) and from the mathematical models (top row). The duration of experimental infections presented in this figure depict a minimum percent increase because the duration of infections cannot be measured exactly (see supplemental material S1), but a minimum value can be estimated.

**Figure 7:**
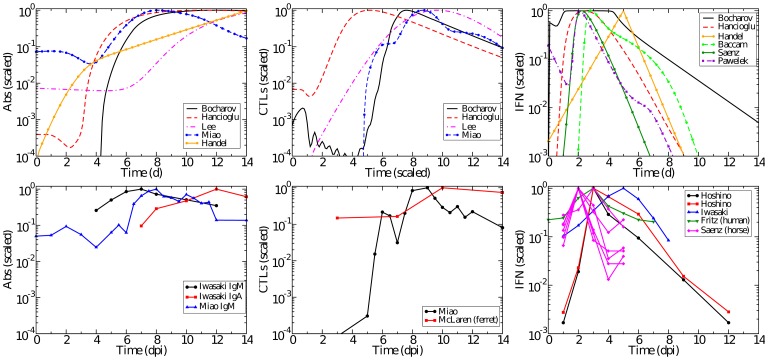
Time course of Abs, CTLs, and IFN. The time courses predicted by models (top row) and experimentally determined time courses (bottom row) for Abs (left), CTLs (centre), and IFN (right). All data has been scaled to peak at 1.0. Experimental data are collected from mice unless otherwise indicated.

In addition to examining the models' predictions of the infection process in the absence of various immune components, we also consider whether the models accurately replicate the time course of the immune response itself. Fig. 7 compares the levels of Abs, CTLs, and IFN over the course of an influenza infection observed experimentally to those predicted by the mathematical models.

In the following sections, we'll discuss the effect of Abs, CTLs, and IFN, independently, in terms of their predicted kinetics and respective significance in modulating infection severity according to the various models.

#### Antibodies

In models that consider Abs (Bocharov, Hancioglu, Lee, Handel, Miao), the Abs act by binding to and removing virus. The manner in which the generation and loss of Abs is implemented, however, varies between models. In the Bocharov model, Abs are produced by plasma cells whose formation is determined by B cells and helper T cells. In the Hancioglu model, Abs are produced by plasma cells that have been activated by the presence of antigen presenting cells. In the Lee model, Abs are produced by short-lived and long-lived plasma cells that have differentiated from B cells. All of these models include decay of Abs; the Bocharov and Hancioglu models also include loss of Abs due to binding with virus. The Miao model uses actual Ab levels from influenza-infected mice. The Handel model neglects Ab decay and assumes Abs grow proportionally to both viral titer and their own concentration, with the latter mechanism meant to emulate clonal expansion of Ab-generating B cells, which are not explicitly represented in the model.

The Ab time courses as predicted by each model are shown in Fig. 7 (left column) along with experimentally determined Ab time courses for comparison. It is interesting that several of the models predict a much greater fold change in Abs over the course of the infection than is actually observed experimentally [11], [23]. Only the Lee and Miao models match the 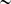
20-fold increase between 4–7 dpi that is seen in experiments. Abs in the Handel model begin at very low levels and appear to be rising too slowly. Abs in the Hancioglu and Bocharov models also begin at low levels, but they rise abruptly around 3–4 dpi.

It is of particular interest to compare the predictions of the Miao split and Miao full models. These models differ only in their fitting procedure; the equations and Ab data are the same. However, the two models predict vastly different effects for Ab removal on the course and severity of influenza infection (Fig. 4, second row). In fitting the Miao split model, we assumed there is no immune response in the first days of the infection, since the data indicates the immune response up to that point is small. The Miao full fit includes the immune response over the entire time course. It is clear that even if the immune response is small during that initial time frame, this initial kinetics difference can have a significant impact on the predicted later time course of the disease.

Despite differences in the Abs and viral kinetics predicted by the various models, all the models are mostly consistent on the predicted effect and importance of Abs in the control of the infection. The models suggest Abs are the most important in reducing the duration of the infection and viral titer AUC (Fig. 6, right). The experimental data suggest that Abs substantially reduce the peak viral titer and viral titer AUC, while IgM and IgG antibodies shorten the duration of the infection. While most models predict that removal of Abs has little effect on the initial peak viral titer (Fig. 5, second row, left), consistent with experimental data, the Handel model, not surprisingly, best reproduces the Iwasaki data [11] because it was fit against this data (Fig. 4). The Bocharov model predicts that removal of Abs results in a chronic infection, a feature consistent with chronic infections of immunocompromised patients, but does not produce the increasing viral titers seen in the Iwasaki data [11] (Fig. 4).

Most of the models predict the removal of Abs has little effect on the fraction of infected cells over the course of the infection (Fig. 5, second row, centre), although the Handel model predicts the number of infected cells will have two peaks in the absence of Abs. Most of the models also predict Abs are not effective in protecting the epithelium by decreasing the fraction of cells infected and killed by the infection (Fig. 5, second row, right, and Fig. 6, right column) with the exceptions again being the Handel and Bocharov models. The Handel model predicts the removal of Abs will result in 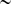
95% of susceptible cells being killed, whereas only 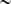
20% are killed when Abs are present; this is because the rapid generation of Abs greatly reduces the viral titer resulting in a less severe infection. In the Bocharov model, whereas in the presence of Abs the epithelium recovers and is fully regenerated, their absence results in a chronic infection with continuous involvement of 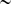
60% of the epithelium.

The results of our assessment of models incorporating Abs are rather mixed. We find that while the Handel model most faithfully reproduces the experimental predictions of viral titer time courses in the presence and absence of Abs, it does not accurately capture the dynamics of the Abs themselves. The Lee model provides a better representation of the time course of Abs and the Miao model uses experimentally measured Ab time courses, although neither model properly replicates the effect of Abs on infection kinetics.

#### Cytotoxic T lymphocytes

Models that incorporate CTLs (Bocharov, Hancioglu, Lee, Miao) implement the action of CTLs as binding to and removing infectious cells. Again, the implementation of the growth and decay of CTLs in different models causes differences in the predicted dynamics. The Bocharov model assumes that the growth rate of CTLs is proportional to the population of antigen presenting macrophages and helper T cells. The Hancioglu model assumes it is proportional to the population of antigen presenting cells and other CTLs. In both models, CTLs are removed either through binding with an infected cell or decay at a constant rate. The Lee model assumes that activation, proliferation, and decay of CTLs is proportional to availability of mature dendritic cells, with no loss of CTLs due to binding with infected cells. The Miao model uses data collected from influenza infections of mice directly in their model rather than modelling CTL kinetics (growth and decay) as an additional equation.

The CTL kinetics time course in Miao [23] indicates a sharp rise of CTLs between 5–8 dpi with decay occurring in roughly three stages: a sharp drop within 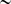
1–2 days followed by a slower decay over the next 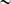
4–5 days and a much slower drop to pre-infection levels over the next week (Fig. 7). A second experimentally determined time course [24] shows a smaller change in CTLs, with a rise in CTLs occurring between 7–10 dpi. Two of the models, Hancioglu and Lee, predict the appearance of CTLs somewhat earlier, around 4 dpi.

Despite differences in CTL and viral kinetics between models, all consistently predict CTLs have little or no effect on the course of the infection (Fig. 5, third row, and Fig. 6, centre column). When effective, as in the Lee and Hancioglu models, they act to mildly decrease infection duration and viral titer AUC. However, removal of the action of CTLs in the models does not lead to sustained, high viral titer or dramatically reduced viral clearance in contrast with what is typically observed experimentally (Fig. 2, centre column). While the models predict little change in the severity of the infection when CTLs are removed, the experimental data suggest that CTLs can substantially reduce viral titer peak, symptomatic duration and viral titer AUC. This discrepancy likely arises because the experimental data against which these models were fitted were insufficient to characterize both the Abs and CTLs' effects independently, causing the parameter fitting routines to ascribe the effect of the combined Ab and CTL response to just Abs. The relatively minor role for CTLs in the models may also be due to the fact that CTLs rise to significant levels only late in the infection process (6–8 dpi). Thus CTLs would not play a role in arresting the infection, but instead would ensure it is cleared effectively to prevent reinfection of newly regenerated epithelium which, if re-infected, could fuel a chronic infection like those established in immunocompromised patients. None of the models predict the establishment of a chronic infection in the absence of CTLs.

#### Interferon

The immune component that has the most varied model implementations is IFN (Fig. 3). Although IFN is known to have many antiviral effects [78]–[80], [84], it is unclear which effect has the most impact on the course of the infection and should be included in a model. In the Bocharov model, IFN is produced by IFN-secreting macrophages and disappears via nonspecific clearance and absorption by target cells causing the latter to become resistant to infection. This protection wanes over time at a constant rate and these uninfected, protected cells gradually become susceptible to infection once again. In the Hancioglu model, IFN also confers a resistance to infection that slowly decays over time. IFN in the Hancioglu model is produced by both infected cells and antigen presenting cells and disappears via absorption by uninfected cells and clearance at a constant rate. In the Saenz model, IFN has a similar action but cells protected by IFN first become partially resistant and can either become permanently resistant or become infected. In the Saenz model, IFN is produced by infected cells and by latently infected, partially resistant cells and decays at a constant rate. The Pawelek model was proposed as an improvement to the Saenz model and was fit to the same data. In the Pawelek model, IFN is produced by infected cells and causes target cells to become resistant to infection. The resistance wanes over time and the resistant cells will eventually become susceptible to infection. Additionally, IFN stimulates production of natural killer (NK) cells which target and kill infected cells. While NK cells are not explicitly included in the model, their effect is assumed to be proportional to the amount of IFN that stimulated their growth. In the Handel model, IFN grows at a rate proportional to the viral load and decays at a constant rate. IFN in the Handel model reduces the production rate of virus by infectious cells, similar to the manner in which the action of neuraminidase inhibitors is typically implemented in these models [4], [7], [8], [90]. In the Baccam model, IFN reduces the viral production rate as in the Handel model, but also acts to lengthen the duration of the eclipse phase in newly infected cells. IFN in the Baccam model grows at a rate proportional to the number of infected cells with a delay of half a day and decays at a constant rate.

IFN kinetics are somewhat consistent across experiments, peaking typically around day 2–3 post-infection, except in [11] where it peaks at 5 (Fig. 7, right column). With the exception of the Bocharov model, the models generally agree with the experimental time course for IFN concentration. The Bocharov model predicts a dramatic rise in IFN almost immediately upon infection, after which IFN remains constant from 1–4 dpi until the infection begins to resolve. And because the Bocharov model predicts IFN levels remain constant over much of the infection, IFN does not contribute in a measurable way to the infection dynamics of that model.

The models (Bocharov, Hancioglu, Handel, Saenz, Pawelek, Baccam) consistently predict IFN confers significant protection of the respiratory tract. Suppressing the effect of IFN in the models leads to far larger numbers of dead cells (Fig. 5, bottom right, and Fig. 6, upper right), with the exception of the Baccam model in which all cells are consumed irrespective of IFN levels. The effect of IFN on the viral titer time course is mostly consistent across the different models (Fig. 5, bottom left), with its absence leading to viral titers peaking later and at higher levels resulting in a longer-lasting, more severe infection with a higher viral titer AUC (Handel, Saenz, Baccam, Pawelek). This is consistent with experimental infections that examine the effect of IFN and predict that absence of IFN leads to a higher viral titer peak, longer-lasting infection with a larger AUC (Fig. 6, bottom right). The Pawelek model depicts an interesting variation of these general dynamics; while a lack of IFN increases the duration of symptomatic duration of the infection (the time when viral titer is greater than 10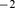), the duration of the entire infection actually decreases in the presence of IFN. The presence of IFN in this model causes a plateau in the viral titer due to waning resistance causing low levels of virus to linger. In the Hancioglu model, however, suppression of IFN actually leads to a more subdued infection of shorter duration and smaller viral titer AUC (Fig. 4). This is due to the protection conferred to uninfected cells by IFN: as IFN levels decay and protection wanes, these cells become available for infection at a time when most susceptible cells have already been consumed. As these cells progressively re-enter the depleted pool of susceptible cells, they rekindle the infection, allowing it to go on a little longer. Suppressing IFN in the Hancioglu model removes this partial protection and allows the infection to proceed through all susceptible cells without delay and resolve more rapidly. This does not occur in the Saenz model because IFN protection in that model does not wane.

The higher, delayed viral titer peak predicted by the models when the action of IFN is suppressed is similar in the experimental suppression experiments (Fig. 2, right column vs Fig. 5, bottom left and Fig. 6, right column), but no model reproduces the longer-lasting, sustained titers observed experimentally when IFN is suppressed. Overall, the Handel, Saenz, Pawelek and Baccam models all fare equivalently well when confronted with viral titer data — they all display a higher viral titer peak and longer infection duration in the absence of IFN, consistent with experimental observations. If the extent of respiratory tract damage could be measured in IFN suppression experiment, it would be possible to test these models' prediction regarding IFN's protective role.

### Neuraminidase inhibitor treatment

An important application of within host models of in vivo infections is to evaluate and optimize antiviral therapies; models can be used to extract the efficacy of antiviral treatment from patient viral titer time courses or to simulate a broad range of doses to determine the best treatment protocols prior to clinical trials. Models of viral decay rates for chronic infections with the human immunodeficiency virus (HIV) or the Hepatitis B and C virus under antiviral therapy have provided key insights into the kinetic parameters driving the infection and the efficacy of the therapy [35], [92], [93].

Unfortunately, several factors prevent within host models from playing a bigger role in antiviral therapy optimization for influenza. One such factor is the acute, short-duration nature of influenza infections. Viral titers from uncomplicated influenza infections are detectable for no more than 4–8 d. Unless samples are taken at least daily, one can at best hope to obtain 8 time points which are often insufficient to characterize the widely varied viral kinetics across individuals over that period. Another important obstacle has been the absence of a valid within host model to capture the kinetics of severe influenza infections characterized by more sustained viral titers. Indeed, mathematical models provide a reasonable fit to viral kinetics from acute, uncomplicated influenza infections, but none can believably reproduce the chronic infections observed in immunocompromised patients or the longer lasting infections seen in children and/or in patients infected by novel, pandemic strains. This weakness is an issue since it is primarily these cases that will require antiviral therapy, whereas therapy is not typically administered in uncomplicated cases. A major obstacle to developing such models is the lack of knowledge and factual, quantitative evidence as to what factor(s) are responsible for the more severe, longer-lasting infections. At this time, possible culprits include cell tropism, lack of pre-existing immunity, and hypercytokinemia. But the relative role of each of these factors has not been elucidated.

Using the models described above, we can investigate the prediction of each model regarding the efficacy of treatment with neuraminidase inhibitor (NAI), both in the presence and absence of the immune responses that are implemented in each model. NAIs inhibit release of newly produced virus from the surface of the infected cells that produced them. Since viral release is not explicitly represented in any of the mathematical models surveyed here, the action of NAIs in all models is implemented as a reduction in the rate of virus production by infected cells [94], [95], such that: 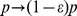, where 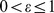 is the antiviral efficacy and represents the fraction of inhibition achieved. Prior to initiation of antiviral therapy 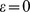, and for simplicity it is instantaneously set to the desired value and held constant thereafter for the remainder of the infection. Note that antiviral efficacy, 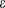, can be related to physiological antiviral concentrations through use of a PD model relating effect to concentration of drug in the plasma, for example, an 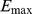 model [96]. Patients typically seek treatment shortly after symptom onset, which typically occurs 1–2 dpi [20], [39]. Therefore, in all models, with the exception of the Handel model, treatment is initiated when the viral titer crosses the symptomatic threshold, i.e. 1% of the peak viral titer. In the Handel model, we initiate treatment at 36 hpi because the initial viral titer in this model is higher than the symptomatic threshold we defined. We use a drug efficacy of 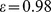, as estimated for NAIs in previous modelling efforts [4], [7], and examine the effects of NAI treatment in the presence of a full immune response as well as in the absence of various immune responses.

**Figure 8:**
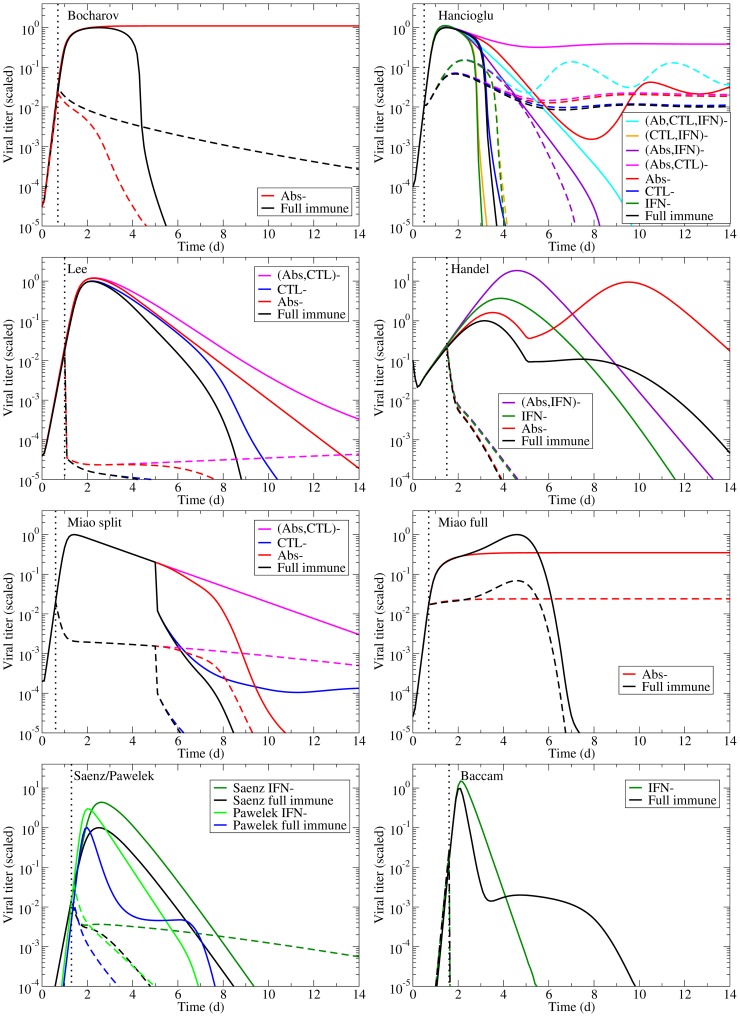
Simulations of the effect of NAI treatment on viral titer. The effect of NAI treatment on the course of influenza in various within host influenza models in the presence of the full immune response and in the absence of various immune components. The vertical dotted line indicates the time of treatment initiation. Inhibition of viral production is assumed to be 98%.

Most of the models predict treatment with NAIs results in a rapid decay of the viral titer (Fig. 8), yielding a reduced viral titer peak and a shorter infection. This behaviour is consistent with what is typically observed in treated patients [39], [97]. A glaring exception is the Hancioglu model which predicts that although NAIs will lower the peak viral titer, they will also increase the duration of the infection because of the protective action of IFN. When IFN therapy is applied in the Hancioglu model, it slows infection progression such that IFN-protected cells lose their resistance before the infection has been effectively cleared and become infected. This coupled with target cell regeneration leads to sustained infections in the presence of NAIs.

**Figure 9:**
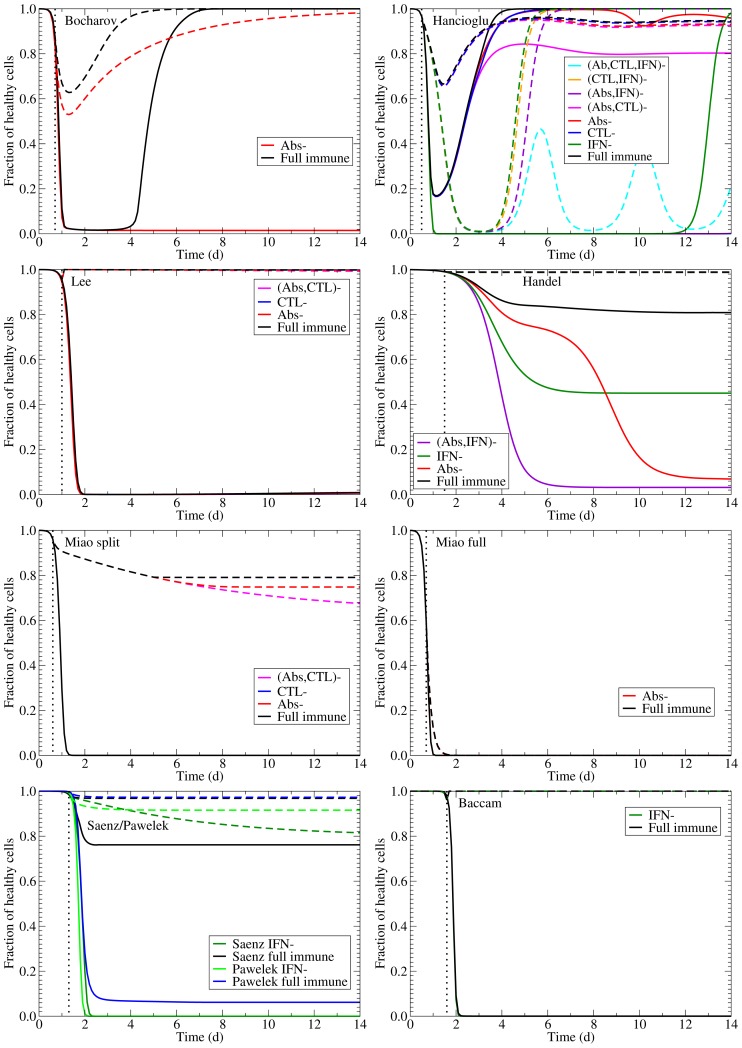
Simulations of the effect of NAI treatment on susceptible cells. The effect of NAI treatment on the course of remaining healthy cells in various within host influenza models in the presence and absence of immune responses. Inhibition of viral production is assumed to be 98%.

A further measure of a drug's ability to minimize illness is the degree to which it confers protection to susceptible cells and limits damage. The effect of NAIs on the fraction of uninfected cells (including those protected by IFN) untouched by the infection is shown in Fig. 9 in the presence of the full immune response and in the absence of various immune components. Most of the models predict NAIs will provide significant protection of the epithelium from damage. The most striking of these are the Baccam and Lee models, which predict almost complete death of the epithelial layer for untreated infection and almost complete protection with NAI treatment. The exceptions are again the Hancioglu and Miao models. The Hancioglu model predicts a longer-lasting infection with NAI treatment that leads to greater cell death, while the Miao model predicts a target cell limited infection even in the presence of NAIs.

## Discussion

### Approach to assessing models

Mechanistic models are valuable tools in identifying the key players of the immune response against an infection and in resolving their mode of action and efficacy [98]. In constructing a mathematical model for the course of an influenza infection within a host, one carefully considers which host factors to include in the model, which to reduce to a simpler reaction, and which to omit [99]. Each mechanism is implemented in the model based on current knowledge regarding host-virus interactions. The act of engineering the host-virus interactions into a model enables us to identify which postulated mechanisms behave inappropriately when implemented [100] and to distill the model down to only those immune response components that are essential to reproducing general infection kinetics.

The approach used here was aimed at assessing strengths and weaknesses of currently available models of influenza infection. Models have two sources of error that must be considered, parameters and structure [101]. One way to assess the adequacy of a mechanistic model is to compare model predictions with experimental data that were not used for estimating parameters in a process called validation aimed at determining whether a model can be appropriately used for its intended purpose [102], [103]. Regardless of how well model simulations match the data used to derive the model parameters, if the model cannot reproduce data from other experiments, then it can be concluded that the model structure or parameters are inadequate. We use the commonly used approach of visual inspection to qualitatively determine the ability of the model to reproduce the shape of the time course data of viral titers and immune response. The mechanistic basis of a model that consistently reproduces the general trend of the data under different conditions (e.g., whether viral titer goes up or down or does not change in response to a component of the immune response turning on or off) can be viewed with more confidence than that of a model that agrees with data only under certain conditions or that fits only a portion of the data (e.g., viral titer in the first two days) perfectly.

Our purpose is to gain insight into the role the immune system plays in influenza infections and to determine whether current mathematical models of influenza infection are consistent with available data to develop a predictive human model. Most of the data used in the assessment were preclinical and while we cannot expect human viral and immune kinetics to match preclinical data exactly, the trends are expected to be similar. Likewise, one would not expect a model developed from data for a given influenza strain in a specific type of mouse to perfectly predict infection dynamics in a different mouse type or for a different influenza strain, but it should qualitatively capture key features of infection kinetics. Therefore, we focus here qualitatively on whether an immune response causes an expected outcome in the model, instead of performing a quantitative comparison.

### Implications of species differences

We must be careful when extrapolating results from animals to humans. Most of the preclinical data presented here comes from mice, whose immune systems differ in some significant ways from humans. Several mouse and human antibody classes differ [28], [29], particularly IgG antibodies, which appear to have evolved independently in humans and mice [104] such that they have different subclasses and functions. There are also differences in the role of type I IFN [105], [106]. In humans, type I IFN provides a direct link between the innate and adaptive immune responses by stimulating growth of Th1 cells. In mice, however, this signalling pathway is not functional and growth of Th1 cells is stimulated by Il-12 [106]. The time course and function of CTLs appears to be similar in humans and mice for a primary infection [27]. In mice, however, CTLs provide heterosubtypic immunity to infection, which is not the case in humans who typically only have subtype-specific immunity [107]. While these are important differences, many of the models considered here do not include such specific details. Current models are still trying to reproduce the general features of influenza infection and the immune response. As mathematical models become more detailed, these species differences will need to be considered.

Of additional concern, mice are not natural hosts for influenza and influenza viral strains sometimes need to be passaged several times in mice before adapting to their new host [108], which leads to changes in the RNA of the virus [109], [110]. These viral genetic changes can lead to changes in host immune response and viral dynamics [108], [110] making it difficult to extrapolate parameters obtained from experiments in mice to dynamics in humans.

### Limitations in available data

The preclinical experimental data we have gathered provides a general picture of the role of Abs, CTLs and IFN. Experiments in which a specific component of the immune response is suppressed offer important insight into the role of individual components of the immune response. Although the data are limited, they suggest that CTLs emerge late in a primary infection and contribute mainly to clearing the infection; that Abs emerge sometime between 4–6 dpi and play a significant role in the resolution of the infection; and that IFN emerges early, disappears rapidly, and typically reduces the peak viral titer. Unfortunately, the sparsity of the data leaves an incomplete picture.

In many of the experiments, we do not know whether the immune component was completely suppressed as it was not measured. In some experiments, viral titer data are not sufficient for ascertaining whether the full range of effects of the suppressed immune component has been captured. In the case of Abs, we found only one experimental study, and while the experiment itself was thorough, corroborating data from similar experiments would make a stronger case for the role of Abs.

Finally, some of the data are contradictory and need further investigation. For example, the data from [68] show little to no increase in the peak viral titer when IFN is removed, while data from [25] and [13] show a clear change in viral titer peak. This apparent discrepancy could be due to a number of factors, including insufficient data in the face of inter-host variability; an insufficient suppression of the full range of action of IFN when its action is suppressed only via STAT1 disruption; or the variability of the IFN response induced by different strains.

### Limitations in model structures

Despite the sparse data and the limitations of animal models, we gleaned a general picture of the role of various immune components and could thus test the accuracy of mathematical models that include an immune response to influenza. As was done in experiments, we systematically turned off the effect of each of the immune components in the models. While all the models assessed here were originally fit to experimental data and thus correctly reproduce some facet of influenza infections, our simple test produced surprising, and sometimes unrealistic, predictions.

Primary influenza infections (e.g., with novel strains) typically last longer than secondary infections due to the delayed or weak adaptive immune response mounted against them [9], [10]. Immunocompromised patients can shed virus for weeks or even months [43]–[60]. It is important that mathematical models be able to reproduce infection dynamics in this vulnerable population to enable exploration of optimal strategy for infection control in these patients. Unfortunately, our analysis finds that only one model is capable of producing a long-lasting or chronic infection in the absence of an immune response, as well as shorter infections in the presence of a full, competent immune response; the Bocharov model is the most realistic model in this respect. Some of the models (Baccam, Saenz, Pawelek) cannot simulate a sustained infection because they do not include cell regeneration. Other models (Handel, Lee, Miao) do not produce sustained infections possibly because suppressing the immune response explicitly included in these models does not correspond to complete suppression: immune components that are not explicitly modelled are implicitly present in other model parameters. For example, in a model without explicit Ab response, data fitting will compensate by setting the viral clearance rate to a larger number to implicitly account for this loss.

Although the Bocharov model correctly produces a chronic infection in the absence of the full immune response, it predicts that suppressing CTLs and/or IFN will have no effect on viral kinetics. This is particularly unfortunate given that this model implements in detail the processes responsible for the production of CTLs and IFN with a large, detailed set of delay differential equations, all of which contribute nothing to shaping viral kinetics, at least for the parameter values reported therein. The Lee model, which is even more complex, also shows very little difference in viral kinetics when CTLs and Abs are turned off, which suggests that perhaps the most important elements driving the infection are lost in all the detail. Other models show a more varied response when various immune components are turned off. The Hancioglu model in particular can produce a wide range of behaviours, from short infections to chronic infections with oscillating viral titers. Unfortunately, these titers do not reflect what we see in the experimental data, particularly with regards to their implementation of IFN. In their model, removal of IFN actually increases the duration of the infection, often turning short infections into chronic infections.

The simpler models, in spite of the smaller number of parameters and equations, can also show interesting dynamics when immune components are turned on and off. The Pawelek, Baccam and Handel models predict a bimodal viral titer in the presence of IFN and a single peak in the absence of IFN. Unfortunately, this behaviour, although sometimes seen in other experiments [4], [111], was not seen in available experimental data presented herein.

There are some limitations to the models presented here. All the models assume a homogeneous target cell population, at least before IFN creates a second class of less susceptible or resistant cells. Dobrovolny et al. [90] showed that the assumption of a heterogeneous cell population could lead to sustained viral titers under certain conditions. While their model did not include an immune response, it would be valuable to understand the role of the immune response under these different infection dynamics. Another limitation in the models discussed here is that they do not link viral titer to clinical outcomes. Recently Canini and Carrat [112] developed a model of influenza infection that included an explicit description of the innate host response and the relationship to clinical symptoms. This model was based on a population analysis of viral kinetics and symptom dynamics data in 44 patients in placebo groups inoculated with H1N1 (A/Texas/91) influenza that had virus-positive nasal wash samples. This model was not included in our analysis because it was published after the work here was completed. It seems likely that limited data may have caused similar issues to those of the models described here. The model was based on viral titers in nasal washes and composite symptom scores; data on the immune response were not included in the study. However, this work made an important step forward in linking the viral titer and immune response to clinical symptoms, and the addition of a drug effect would take it another important step forward.

### Recommendations for further experiments

The ideal experiment for deducing the role of a particular immune component requires comprehensive collection of data. For example, Iwasaki et al. created mice with different immune components knocked out (Abs and/or CTLs and/or IFN); they infected the mice (and their normal counterparts) and measured not only the viral titer, but also the fraction of dead cells, Abs and IFN over time [11]. The only measurement that was missing was CTLs. Viral titer from normal and knock-out animals show the manner in which the immune component affects the infection time course. Importantly, time course measurements of Abs, IFN, and CTLs make it possible to verify that the immune component under study was entirely eliminated, and that the other immune components were not affected. Changes in viral titer can be correlated with changes in the levels of specific immune components.

Another experiment that would be helpful is one where the “dose” of a particular immune component could be controlled and varied. In dose ranging experiments, usually performed for antiviral drugs [5], different doses of Abs, CTLs, or IFN would be applied to in vitro influenza infections and the time course of viral titer be measured. These data can be used to test which hypothesis, or mathematical implementation, for the role of Abs, CTLs, or IFN best explains the infection dynamics. This type of dose ranging experiment might be particularly useful in teasing out the role of CTLs, which appear not to play a large role in primary infections, but play a larger role in preventing or mediating a secondary infection [15]. Experiments have shown that CTLs can be effective at reducing viral loads if they appear early enough during the infection [73], suggesting that it would be easier to isolate their effect if studied in this context.

While the data presented here are a good start to these types of experiments, a systematic set of experiments, with well-sampled, complete time courses of all measured components, is needed to help construct a model that correctly captures the dynamics of the immune response. It is particularly important that the time course be sampled frequently and for the full duration of the infection (viral titer data from Seo et al. [13] is a good example), so that biologically relevant parameters can be determined from the data.

There are practical limitations that must be considered in designing such experiments. Frequent sampling of human subjects might deter patients from participating in studies. It is difficult to study viral and immune kinetics at the actual site of infection in humans. Viral load is typically measured by collecting nasal washes or nasal swab. We know, however, that viral kinetics of nasal swabs differ from viral kinetics of tracheal aspirates [113]. The human immune response is typically measured by taking blood samples, which is problematically invasive, but potentially remedied by measuring levels of various immune components in nasal wash [20]. It is unclear how levels of immune components in serum or nasal wash are related to levels at the site of infection, although antibody levels in serum and tracheobronchial secretions are known to differ substantially [114], [115]. For these reasons, it is more practical to perform experimental studies in animal models.

## Conclusions

In this paper, we examined preclinical and clinical studies of the role of the immune response to formulate key features of an immune response that should be captured by mathematical models. We tested existing mathematical models that incorporate an immune response to see if they qualitatively reproduce important features of the immune response. An attempt to validate the models using this general picture synthesized from multiple types of data indicates that the models examined here fail to accurately reproduce at least one aspect of the immune response, even though the model parameter values are derived by fitting experimental data. We hope this analysis will stimulate more comprehensive experiments into the role of the immune response in influenza infections and that the approach presented here will be useful for building confidence in new models moving forward.

## Methods

### Experimental data

Literature for preclinical or clinical data on the role of the immune response during an influenza infection was found by searching the Pubmed and Web of Science databases using the keywords “influenza” and one of “antibodies”, “interferon”, or “cytotoxic T lymphocytes” to find studies of the dynamics of these key immune components during influenza infection. We examined the search results for experimental studies that measured the levels of Abs, CTLs or IFN during an influenza infection, or experiments that measured viral titer when one of these responses was suppressed. We extracted data from figures in those papers that had data for three or more time points during the infection. Table 3 lists the papers from which we extracted experimental data, the figure from which data was extracted, and the type of data extracted from each paper. We also searched the same two databases using the keywords “influenza” and “immunocompromised.” Table 1 is the resulting list of papers that report cases of immunocompromised patients infected with influenza. Details of their underlying medical conditions, the strain of influenza with which they were infected, and the antivirals used to treat them are tabulated. When possible, we extracted viral time course data for immunocompromised patients. Unless otherwise noted, we used Engauge Digitizer 4.1 [116] to extract the data.

**Table 3:** Summary of experimental data presented in this paper.

Paper	Figure(s)	Animal	Strain 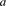	Sampling site	Type of data
Iwasaki [11]	1,7	mouse	PR8	lung homogenates	Viral titers with and without Abs
Neff-LaFord [70]	1	mouse	HKx31	lung homogenates	Viral titers with and without CTLs
Kris [66]	2 (lung)	mouse	PC73	lung homogenates	Viral titers with and without CTLs
Wells [67]	2A	mouse	PC73	lung homogenates	Viral titers with and without CTLs
Yap [69]	2	mouse	WSN33	lung homogenates	Viral titers with and without CTLs
García-Sastre [68]	1	mouse	PR8,WSN33	lung homogenates	Viral titers with and without IFN
Hoshino [25]	2	mouse	PR8	lung homogenates	Viral titers with and without IFN
Seo [13]	2a	pig	PR8	nasal swabs	Viral titers with and without IFN
Iwasaki [11]	2	mouse	PR8	tracheobronchial wash	Time course of IFN and Abs
Miao [23]	2b,d 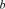	mouse	HKx31	lung homogenates	Time course of Abs and CTLs
McLaren [24]	3	ferret	PC73	lung wash	Time course of CTLs
Fritz [20]	5	human	TX91	nasal lavage	Time course of IFN- 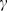
Hoshino [25]	3b	mouse	PR8	lung homogenates	Time course of IFN

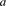
 Full names of the viral strains are A/Puerto Rico/8/1934 (H1N1) (PR8), A/Hong Kong/X31 (H3N2) (HKx31), A/Port Chalmers/1/73 (H3N2) (PC73), A/Wisconsin/1933 (H1N1) (WSN33), A/Texas/36/91 (H1N1) (TX91).

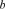
 Data was not extracted from the paper, but was provided to us by Hongyu Miao.

### Mathematical models

We searched the literature for in-host models of influenza that incorporate at least one component of the immune response and whose parameters were determined, at least in part, from experimental data. We found eight models that fit our criteria: Bocharov and Romanyukha [2], Hancioglu et al. [31], Lee et al. [22], Handel et al. [6], Miao et al. [23], Saenz et al. [32], Pawelek et al. [37] and Baccam et al. [4]. The models are summarized in Table 2, and described in more detail in supplemental material S2, which also contains model equations.

For each model, with one exception, we use the parameters determined in the original paper. Miao et al. used two different methods for fitting their model to their data. First, they included the immune response from the start of the infection (the Overall parameters in Table 2 of their paper). We refer to their model using Overall parameters as the Miao full model. Second, they explicitly assumed the immune response did not play a role during the first 5 days of the infection by setting all parameters associated with the immune response to zero for the first 5 days (the Early and Adaptive parameters in Table 2 of their paper) [23]. Miao et al. fitted their Early parameters using viral titer data up to day 5 post-infection and setting all immune response parameters to zero (i.e., assuming the immune response up to that point is negligible). They fitted their Adaptive parameters by using the viral titers from day 5 onward leaving all parameters free. This resulted in two sets of parameters: the Early parameters for infection kinetics prior to day 5 and the Adaptive parameters representing the kinetics after day 5.

It is possible parameters such as the rate of cell infection by virus or the infectious cell lifespan change drastically as the immune response emerges, perhaps through the action of cytokines which are not explicitly represented in their model. The Early and Adaptive parameters suggest the arrival of the immune response leads to an increase in cell infection rate and in the infectious cell lifespan, which is inconsistent with the action of immune-mediated cytokines. Therefore, we developed an alternative fit that we refer to as the Miao split model. The Miao split parameters were fit by using their viral titer data, but up to day 5 post-infection all immune system parameters were set to zero (as was done for the Miao et al. Early parameters), and from day 5 onward all infection parameters were fixed and only the parameters associated with the immune response varied freely. The new Miao split model parameters are listed in Table 4.

**Table 4:** Parameter values of the Miao split model using the Miao et al. model and data [23].

Description*	Parameter	Value
Infectivity of virions	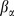	2.5 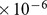 mL⋅ 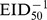 ⋅d^−1^
Target cell regeneration rate	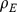	1.2 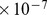 d^−1^
Death rate of infectious cells	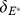	0.47 d^−1^
Viral titer clearance rate	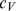	6.9 d^−1^
Viral production rate by infectious cells	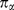	100 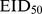 ⋅mL^−1^⋅d^−1^ (fixed)
Initial number of susceptible cells	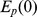	6.5 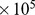
Initial virus concentration	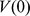	1473 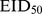 ⋅mL^−1^(fixed)
Killing rate of infectious cells by CTLs	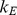	5.6 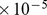 d^−1^
Removal rate of virions by IgG Abs	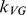	3.0 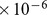 mL⋅(pg⋅d)^−1^
Removal rate of virions by IgM Abs	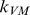	6.5 mL⋅(pg⋅d)^−1^

* See supplemental material S2 for a description of this model.

In order to easily compare model predictions, all models have been scaled so their viral titer in the presence of their full immune response arbitrarily peaks at a value of 1. We chose to scale the models in the presence of their full immune response because it is in this condition that each model was fit against experimental data. The scaling factors, 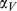, were calculated from the viral titer peak, 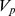, such that 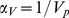 for each model and were used to scale all variables and parameters containing units of viral titer. For example, model parameter 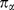 in Table 4 which has units of EID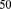/mL/day, would be scaled such that 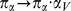. All time course data produced from a mathematical model and presented herein are scaled in this manner. Note that although this scaling changes how high the viral titer peaks, it does not change the dynamical behaviour of the model or the shape of the curves.

Models were simulated using lsode for ordinary differential equations or dde23 for delay differential equations in Octave 3.0.5 [117]. Individual immune components were turned off by setting the parameter that controls their effect on cells or virus to zero. For example, the effect of CTLs is often modelled using the term 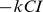 in the differential equation for infected cells where 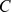 are the CTLs, 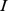 the infected cells, and 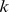 the rate at which CTLs kill infected cells. To determine the effect of turning off CTLs, we set 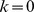, leaving the differential equation for CTLs untouched.
