# Correction: Assessing Mathematical Models of Influenza Infections Using Features of the Immune Response

**DOI:** 10.1371/journal.pone.0099740

**Published:** 2014-06-02

**Authors:** The PLOS ONE Staff

Two Supporting Information files were mistakenly omitted. Please view these Supporting Information files.

## Supporting Information

File S1 Infection Severity
(PDF)

File S2 Model Equations
(PDF)
