# Supplementary material for: Correction: Assessing Mathematical Models of Influenza Infections Using Features of the Immune Response
Source: PLoS One. 2014 Jun 2;9(6):e99740. doi: 10.1371/journal.pone.0099740 (PMC4041882; doi:10.1371/journal.pone.0099740)
Supplement: File S1 — Infection Severity (PDF) [file pone.0099740.s001.pdf]

Determining measures of infection severity for experimental data

Similar to what was done with the mathematical models, we computed three measures to quantify the increase (or decrease) in infection severity when a particular immune component is suppressed: peak viral titer, symptomatic duration, and AUC. The fourth measure considered with the mathematical models, namely the fraction of dead cells at the end of the infection, could not be determined from the experimental data. While calculating these measures for data generated from computer simulations is straightforward, the sparsity of experimental data makes calculating the measures in these data imprecise. To determine peak viral titer of the experimental data, we used the same method as for simulated data — we used the maximum value of viral titer. While this might lead to errors in the estimated values of the peak, some of the data sets have too few points to even fit a simple triangular function to them, which has four parameters, and can be used to determine viral titer peak (1). We define the symptomatic duration as the time spent above the symptomatic threshold (1% of the viral titer peak (2, 3)). For many data sets, we cannot calculate the symptomatic duration because the first time point of the experimental data is already above our defined symptomatic threshold. For data sets where data collection starts early enough, we find the time of crossing the symptomatic threshold by fitting a straight line (to $\log(V)$) to the two pairs of data points on either side of the peak that are above and below the threshold and use the line to interpolate the crossing time. In some cases (for some of the Iwasaki data) we can determine a minimum percent increase because we can calculate the duration of the infection in the presence of a full immune response, but not in the absence

Table I: Measures of infection severity determined from experimental data.

Immune response	Data set	Peak viral titer ([V])			Symptom. Duration (d)			AUC ([V] $\cdot$ d)		
		+	-	% diff.	+	-	% diff.	+	-	% diff.
Abs	Iwasaki, no Ab	7.1×10^5	7.3×10^7	10000	7.1	>8	>12	1.6×10^6	4.4×10^7	2600
	Iwasaki, IgM	7.1×10^5	2.4×10^8	34000	7.1	>12	>68	1.6×10^6	4.8×10^8	30000
	Iwasaki, IgG	7.1×10^5	1.0×10^6	47	7.1	6.6	-7.2	1.6×10^6	2.4×10^6	49
	Iwasaki, IgM	7.1×10^5	1.0×10^7	1400	7.1	>19	>40	1.6×10^6	1.4×10^7	780
CTLs	Neff-LaFord	4.9×10^4	5.1×10^4	3.5	–	–	–	1.8×10^5	2.2×10^5	17
	Kris	1.1×10^5	6.7×10^5	680	–	–	–	5.3×10^5	7.2×10^6	1300
	Wells	3.2×10^6	1×10^6	-69	4.8	12	140	1.2×10^7	5.9×10^6	-49
	Yap	3.5×10^6	1.4×10^7	310	–	–	–	1.4×10^7	5.3×10^7	270
IFN	Garcia-Sastre PR8	1.6×10^4	3.1×10^4	89	–	–	–	5.7×10^4	6.8×10^4	20
	Garcia-Sastre WSN	9.6×10^9	1.9×10^7	24	–	–	–	1.8×10^7	1.0×10^8	450
	Hoshino	2.6×10^5	1.8×10^6	610	–	–	–	8.1×10^5	6.2×10^6	670
	Seo	6.3×10^4	2.1×10^5	230	2.4	5.6	140	6.7×10^4	6.8×10^5	910

of Abs. However, since the viral titer is always above the symptomatic threshold, we can use this time as an estimated minimum for the symptomatic duration in the absence of Abs. AUC is calculated using the trapezoidal method, i.e. the AUC between two adjacent points is approximated by a trapezoid defined by the two data points and the x-axis. The fraction of dead cells cannot be determined for any of the experiments. The results of our calculations are presented in Table I and Fig. 6 (bottom row).

References

1. Holder BP, Beauchemin CA (2011) Exploring the effect of biological delays in kinetic models of influenza within a host or cell culture. BMC Public Health 11: S10.

2. Dobrovolny HM, Baron MJ, Gieschke R, Davies BE, Jumbe NL, et al. (2010) Exploring cell tropism as a possible contributor to influenza infection severity. PLoS ONE 5: e13811.

3. Dobrovolny HM, Gieschke R, Davies BE, Jumbe NL, Beauchemin CAA (2011) Neuraminidase inhibitors for treatment of human and avian strain influenza: A comparative study. J Theor Biol 269: 234–244.
