# Supplementary material for: Correction: Assessing Mathematical Models of Influenza Infections Using Features of the Immune Response
Source: PLoS One. 2014 Jun 2;9(6):e99740. doi: 10.1371/journal.pone.0099740 (PMC4041882; doi:10.1371/journal.pone.0099740)
Supplement: File S2 — Model Equations (PDF) [file pone.0099740.s002.pdf]

Equations for the models with an immune response

This appendix includes the equations for all models along with a brief description of the model dynamics. Note that in some cases, parameter and variable notation has been changed from the original papers so that it is consistent across all models presented in this paper. For full descriptions of the models, all the processes they describe and definitions of all parameters and variables, see the original papers.

Bocharov

The Bocharov model (1) is the first within host influenza model that incorporates a full immune response.

$$
\begin{aligned}
T &= T_0 - I - D - R \\
\frac{\mathrm{d}I}{\mathrm{d}t} &= \beta TV - k_E IC - \delta I \\
\frac{\mathrm{d}D}{\mathrm{d}t} &= k_E IC + \delta I - \alpha_D D \\
\frac{\mathrm{d}R}{\mathrm{d}t} &= \sigma_F FT - \alpha_R R \\
\frac{\mathrm{d}V}{\mathrm{d}t} &= pI + nb_{IC} IC - \gamma_{VA} VA - cV \\
&- \gamma_{VI} VT \\
\frac{\mathrm{d}M}{\mathrm{d}t} &= \gamma_M M_0 V - \alpha_M M \\
\frac{\mathrm{d}H_C}{\mathrm{d}t} &= b_H^C[\zeta \rho_H^C M(t-\tau_H^C) H_C(t-\tau_H^C) - MH_C] \\
&- b_P^{H_C} MH_C C + \alpha_H^C (H_{C0} - H_C) \\
\frac{\mathrm{d}H_B}{\mathrm{d}t} &= b_H^B[\zeta \rho_H^B M(t-\tau_H^B) H_B(t-\tau_H^B) - MH_B] \\
&- b_P^{H_B} MH_B B + \alpha_H^B (H_{B0} - H_B) \\
\frac{\mathrm{d}C}{\mathrm{d}t} &= b_P^C[\zeta \rho_C M(t-\tau_C) H_C(t-\tau_C) C(t-\tau_C) \\
&- MH_C C] - b_{IC} IC + \alpha_C (C_0 - C) \qquad (1) \\
\frac{\mathrm{d}B}{\mathrm{d}t} &= b_P^B[\zeta \rho_B M(t-\tau_B) H_B(t-\tau_B) B(t-\tau_B) \\
&- MH_B B] + \alpha_B (B_0 - B) \\
\frac{\mathrm{d}P}{\mathrm{d}t} &= b_P^P \zeta \rho_P M(t-\tau_P) H_B(t-\tau_P) B(t-\tau_P) \\
&+ \alpha_P (P_0 - P) \\
\frac{\mathrm{d}M_F}{\mathrm{d}t} &= \gamma_{M_F} V(M_{F0} - M_F) - \alpha_{M_F} M_F \\
\frac{\mathrm{d}F}{\mathrm{d}t} &= \rho_F M_F - \alpha_F F - \sigma_F F(T_0 - I - D - R) \\
\frac{\mathrm{d}A}{\mathrm{d}t} &= \rho_A P - \gamma_{AV} AV - \alpha_A A \\
\zeta(D) &= 1 - \frac{D}{T_0}
\end{aligned}
$$

This model does not explicitly include the dynamics of target cells, but the initial number of target cells is included as T_0. Cells become productively infected (no eclipse phase), I, at a rate β and are either destroyed by CTLs, C, or die after a time $1/\delta$, becoming dead cells, D, in either case. Infected cells produce virus, V, at a rate p, with additional virus being released when CTLs destroy infected cells. Virus is removed at a constant decay rate, c, or via binding to Abs, $\gamma_{VA}A$, or entry into target cells $\gamma_{VI}T$. The virus stimulates macrophages to begin producing IFN M_F or to become antigen presenting cells M. Interferon is produced only by macrophages in this model and causes target cells to become resistant to infection, R, although this resistance can wear off. The antigen presenting macrophages cause proliferation of helper T cells (H_B and H_C) that themselves cause proliferation of CTLs and B cells B. B cells cause proliferation of plasma cells, P, which produce Abs.

Hancioglu

The Hancioglu model (2) is a simplification of the Bocharov model. In this model, target cells, T, are infected and immediately become productive, I. Infected cells are either killed by CTLs, C or die after a time $1/\delta$. Macrophages, M, become activated by the presence of dead cells, D, and virus, V. Macrophages and infected cells secrete IFN, F, which acts to protect target cells by making them resistant to infection, R. Macrophages also cause proliferation of CTLs and plasma cells, P. Plasma cells produce Abs, A, which bind to virus at a rate determined by their antigenic distance, S, from the virus. Virus is produced by infected cells and is removed by Abs, absorption into target cells, decay, or other non-specific

clearance (such as coughing).

$$
\begin{aligned}
\frac{\mathrm{d}T}{\mathrm{d}t} &= \lambda D(T+R) + a_R R - \beta T V - \phi F T \\
\frac{\mathrm{d}I}{\mathrm{d}t} &= \beta T V - k_E I C - \delta I \\
D &= 1 - H - R - I \\
\frac{\mathrm{d}R}{\mathrm{d}t} &= b_{FT} T F - a_R R \\
\frac{\mathrm{d}V}{\mathrm{d}t} &= pI - \gamma_{VA} S A V - \gamma_{VH} V T - cV - \frac{a_{V1} V}{1 + a_{V2} V} \\
\frac{\mathrm{d}M}{\mathrm{d}t} &= (b_{MD} D + b_{MV} V)(1 - M) - a_M M \\
\frac{\mathrm{d}F}{\mathrm{d}t} &= b_F M + c_F I - b_{FT} T F - a_F F \\
\frac{\mathrm{d}C}{\mathrm{d}t} &= b_{CM} M C - b_{CI} I C + a_C (1 - C) \qquad (2) \\
\frac{\mathrm{d}P}{\mathrm{d}t} &= b_{PM} M P + a_P (1 - P) \\
\frac{\mathrm{d}A}{\mathrm{d}t} &= b_A P - \gamma_{VA} S A V - a_A A \\
\frac{\mathrm{d}S}{\mathrm{d}t} &= rP(1 - S)
\end{aligned}
$$

Lee

The Lee model (3) is perhaps the most realistic representation of the adaptive immune response. It consists of 15 equations and 46 parameters.

$$
\begin{aligned}
\frac{\mathrm{d}T}{\mathrm{d}t} &= \lambda(T_0 - T) - \beta T V \\
\frac{\mathrm{d}I}{\mathrm{d}t} &= \beta T V - k_E I \gamma T_E(t-\tau_T) - \delta I \\
\frac{\mathrm{d}V}{\mathrm{d}t} &= pI - cV - k_V V A \\
\frac{\mathrm{d}D}{\mathrm{d}t} &= \lambda_D(D_0 - D) - \beta_D D V \\
\frac{\mathrm{d}D^*}{\mathrm{d}t} &= \beta_D D V - \delta_D D^* \\
\frac{\mathrm{d}D_M}{\mathrm{d}t} &= k_D D^*(t-\tau_D) - \delta_{D_M} D_M \\
\frac{\mathrm{d}H_N}{\mathrm{d}t} &= \delta_{H_N}(H_{N0} - H_N) - \pi_H(D_M) H_N \\
\frac{\mathrm{d}H_E}{\mathrm{d}t} &= \pi_H(D_M) H_N + \rho_H(D_M) H_E - \delta_H(D_M) H_E \\
\frac{\mathrm{d}T_N}{\mathrm{d}t} &= \delta_{T_N}(T_{N0} - T_N) - \pi_T(D_M) T_N \\
\frac{\mathrm{d}H_E}{\mathrm{d}t} &= \pi_T(D_M) T_N + \rho_T(D_M) T_E - \delta_T(D_M) T_E \\
\frac{\mathrm{d}B_N}{\mathrm{d}t} &= \delta_{B_N}(B_{N0} - B_N) - \pi_B(D_M) B_N \\
\frac{\mathrm{d}B_A}{\mathrm{d}t} &= \pi_B(D_M) B_N + \rho_{B_A}(D_M + hH_E) B_A \\
&- \delta_{B_A} B_A - \pi_S B_A - \pi_L H_E B_A \\
\frac{\mathrm{d}P_S}{\mathrm{d}t} &= \pi_S B_A - \delta_S P_S \\
\frac{\mathrm{d}P_L}{\mathrm{d}t} &= \pi_L H_E B_A - \delta_L P_L \\
\frac{\mathrm{d}A}{\mathrm{d}t} &= \pi_{AS} P_S + \pi_{AL} P_L - \delta_A A
\end{aligned}
\tag{3}
$$

Briefly, target cells T are infected at a rate β and are generated at a rate λ. Infected cells, I are immediately productive (no eclipse phase) and can be removed by effector T cells, T_E, or death after $1/\delta$. Virus, V, is removed through non-specific clearance c or by Abs, A. Dendritic cells, D, can also become infected (D^*), but they do not produce virus. Instead

they migrate to the lymph nodes where they become mature dendritic cells, D_M and initiate the adaptive immune response. The activation, π_i, proliferation, ρ_i and clearance δ_i of naive CD4$^+$ cells, H_N, effector CD4$^+$ cells, H_E, naive CD8$^+$ cells, T_N, effector CD8$^+$ cells, T_E, naive B cells, B_N, and activated B cells, B_A, are controlled by the number of mature dendritic cells. Effector CD4$^+$ cells assist in the proliferation of activated B cells and mediate differentiation into long-lived Abs-secreting plasma cells P_L. Short-lived Abs-secreting plasma cells, P_S, arise from activated B cells alone.

Handel

Handel et al. investigated a model that included both the effect of IFN (innate immune response) and Abs (adaptive immune response) (4). This model includes an eclipse phase, and allows for regeneration of dead epithelial cells.

$$
\begin{aligned}
\frac{\mathrm{d}T}{\mathrm{d}t} &= \lambda D - \beta T V \\
\frac{\mathrm{d}E}{\mathrm{d}t} &= \beta T V - kE \\
\frac{\mathrm{d}I}{\mathrm{d}t} &= kE - \delta I \\
\frac{\mathrm{d}D}{\mathrm{d}t} &= \delta I - \lambda D \\
\frac{\mathrm{d}V}{\mathrm{d}t} &= \frac{pI}{1+\kappa F} - cV - \gamma\beta T V - aAV \\
\frac{\mathrm{d}A}{\mathrm{d}t} &= sV + rA \\
\log_{10}(F(t)) &= 0.5388t - 0.08429 \quad t \leq 5 \\
\log_{10}(F(t)) &= -0.7435t + 6.328 \quad t > 5 \ ,
\end{aligned}
\tag{4}
$$

where $F(t)$ (IFN) was determined by fitting IFN levels from mice infected with A/Port Chalmers/1/73 (H3N2). Target cells, T are infected at a rate β and re-grown at a rate λ. Once infected, they move to an eclipse phase E before becoming productively infectious, I, after an average time of $1/k$. Infected cells produce virus at a rate p before dying after an average lifespan of $1/\delta$. Virus, V is cleared from the system through non-specific clearance (at a rate c) or by Abs, A, at a rate a. The Abs grow proportional to virus, at a rate s, and

undergo clonal expansion at a rate r. Interferon reduces the production rate, similar to a drug, with an IC_{50} of $1/\kappa$.

Miao

$$
\begin{aligned}
\frac{\mathrm{d}T}{\mathrm{d}t} &= \lambda T - \beta TV \\
\frac{\mathrm{d}I}{\mathrm{d}t} &= \beta TV - \delta I - k_E I C(t) \\
\frac{\mathrm{d}V}{\mathrm{d}t} &= pI - cV - k_G V A_G(t) - k_M V A_M(t) \ .
\end{aligned}
\tag{5}
$$

In this model, the target cells, T, are infected at a rate βV and are born at a rate λ. Once virus enters the cells, they immediately become productively infectious, I, (no eclipse phase) and can be removed either through natural death at a rate δ or they can be killed by CTLs, C, at a rate $k_E C(t)$. Virus is produced at a rate p and decays at a rate c. Virus can be removed from the system by either IgG Abs, A_G, at a rate $k_G A_G(t)$ or by IgM Abs, A_M, at a rate $k_M A_M(t)$. In their implementation of this model, Miao et al. used data collected from mice to numerically create functions for $C(t)$, $A_G(t)$, and $A_M(t)$ (5).

Baccam

While Baccam et al. present several models in (6) in an attempt to describe patient data, only one includes an immune response.

$$
\begin{aligned}
\frac{\mathrm{d}T}{\mathrm{d}t} &= -\beta TV \\
\frac{\mathrm{d}E}{\mathrm{d}t} &= \beta TV - \frac{k}{1+\epsilon_1 F} E \\
\frac{\mathrm{d}I}{\mathrm{d}t} &= \frac{k}{1+\epsilon_1 F} E - \delta I \\
\frac{\mathrm{d}V}{\mathrm{d}t} &= \frac{p}{1+\epsilon_2 F} I - cV \\
\frac{\mathrm{d}F}{\mathrm{d}t} &= sI(t-\tau) - \alpha F
\end{aligned}
\tag{6}
$$

In this variation of the basic influenza model, target cells, T, are infected at a rate β. Once infected, cells enter an eclipse phase, E, in which they do not produce any virus. The cells

become productively infected, I, after a time $1/k$, a time that is lengthened with in the presence of IFN. Productively infected cells produce virus at a rate p, which is suppressed by IFN. IFN is released by productively infected cells at a rate s and decays at a rate α. The IC_{50} of IFN is $1/\epsilon_1$ for the length of the eclipse phase and $1/\epsilon_2$ for the production rate.

Saenz

In (7), Saenz et al. introduced a within host influenza model that includes an IFN response.

$$
\begin{aligned}
\frac{\mathrm{d}T}{\mathrm{d}t} &= -\beta TV - \phi TF \\
\frac{\mathrm{d}E_1}{\mathrm{d}t} &= \beta TV - k_1 E_1 \\
\frac{\mathrm{d}E_2}{\mathrm{d}t} &= m\beta VW - k_2 E_2 \\
\frac{\mathrm{d}I}{\mathrm{d}t} &= k_1 E_1 + k_2 E_2 - \delta I \\
\frac{\mathrm{d}W}{\mathrm{d}t} &= \phi TF - m\beta VW - aW \\
\frac{\mathrm{d}R}{\mathrm{d}t} &= aW \\
\frac{\mathrm{d}V}{\mathrm{d}t} &= pI - cV \\
\frac{\mathrm{d}F}{\mathrm{d}t} &= nqE_2 + qI - \alpha F
\end{aligned}
\tag{7}
$$

In this model, target cells, T, are either infected (at a rate β) or become resistant to infection, W, because of the presence of IFN. Partially resistant cells can still become infected, although the rate is reduced by m. Once infected, cells move into an eclipse phase, E_1 for unprotected target cells or E_2 for partially resistant cells. Eclipse cells become productively infected, I, after a time $1/k_1$ for E_1 or $1/k_2$ for E_2. Partially resistant cells that are not infected after a time $1/a$ become fully resistant to infection, R. Virus is produced by infected cells at a rate p and cleared at a rate c. Interferon is produced by infected cells at a rate q and by E_2 cells at a rate reduced by n and cleared at a rate α.

Pawelek

Pawelek et al. (8) propose several models that they argue better represent the data presented by Saenz et al. (7). We use the model presented in the main body of their paper,

$$\begin{aligned}\frac{\mathrm{d}T}{\mathrm{d}t} &= -\beta TV - \phi TF + \rho R \\ \frac{\mathrm{d}I}{\mathrm{d}t} &= \beta TV - \delta I - \kappa IF \\ \frac{\mathrm{d}R}{\mathrm{d}t} &= \phi TF - \rho R \\ \frac{\mathrm{d}V}{\mathrm{d}t} &= pI - cV \\ \frac{\mathrm{d}F}{\mathrm{d}t} &= qI - dF.\end{aligned} \tag{8}$$

In this model, IFN has two effects: it provides protection from infection to some target cells T by moving them to a resistant class R and it causes death of infected cells by stimulating growth of natural killer cells (not explicitly represented in the model). Unprotected target cells become infected and move directly to a productively infective state (no eclipse phase). The death rate of infected cells is constant until the adaptive immune kicks in (about day 6 or 7) and then is given by $\delta = \delta_I e^(\sigma(t - \mu))$ where δ_I is the original death rate, μ is the turn on time of the adaptive response and σ is the growth rate of the adaptice response. Growth of IFN in this model is assumed to be proportional to the number of infected cells and IFN decays at a constant rate.

References

1. Bocharov GA, Romanyukha AA (1994) Mathematical model of antiviral immune response III. Influenza A virus infection. J Theor Biol 167: 323–360.

2. Hancioglu B, Swigon D, Clermont G (2007) A dynamical model of human immune response to influenza A virus infection. J Theor Biol 246: 70–86.

3. Lee HY, Topham DJ, Park SY, Hollenbaugh J, Treanor J, et al. (2009) Simulation and prediction of the adaptive immune response to influenza A virus infection. J Virol 83: 7151–7165.

4. Handel A, Longini IM, Antia R (2010) Towards a quantitative understanding of the within-host dynamics of influenza A infections. J R Soc Interface 7: 35–47.

5. Miao H, Hollenbaugh JA, Zand MS, Holden-Wiltse J, Mosmann TR, et al. (2010) Quantifying the early immune response and adaptive immune response kinetics in mice infected with influenza A virus. J Virol 84: 6687–6698.

6. Baccam P, Beauchemin C, Macken CA, Hayden FG, Perelson AS (2006) Kinetics of influenza A virus infection in humans. J Virol 80: 7590–7599.

7. Saenz RA, Quinlivan M, Elton D, MacRae S, Blunden AS, et al. (2010) Dynamics of influenza virus infection and pathology. J Virol 84: 3974–3983.

8. Pawelek KA, Huynh GT, Quinlivan M, Cullinane A, Rong L, et al. (2012) Modeling within-host dynamics of influenza virus infection including immune responses. PLoS Comput Biol 8: e1002588.
